# USING A SOCIOCULTURAL LENS TO EVALUATE CANCER SURVIVORSHIP IN BLACKS AND LATINX IN THE US

**DOI:** 10.1093/geroni/igad104.1936

**Published:** 2023-12-21

**Authors:** Candidus Nwakasi, Darlingtina Esiaka, Saamantha Pierre-Louis, Onyeka Ekwebene

**Affiliations:** University of Connecticut, Storrs, Connecticut, United States; University of Kentucky, Lexington, Kentucky, United States; Providence College, Providence, Rhode Island, United States; East Tennessee State University, Johnson City, Tennessee, United States

## Abstract

**Background:**

A sociocultural lens such as the PEN-3 Cultural Model offers an opportunity to better understand the extreme psychosocial, physical, emotional, and financial challenges of Black and Latinx cancer survivors in the community experience. By focusing on cultural identity, relationships and expectations, and cultural empowerment, the PEN-3 Cultural Model applied in this study aims to offer a unique insight into cancer survivorship in the Black and Latinx communities in the U.S. Method. A qualitative descriptive approach was used on a purposive sample of 46 Black and Latinx participants recruited. Each of the 19 participants who are cancer survivors including 7 Blacks and 13 Latinx (Mage = 62 years), was interviewed using a semi-structured interviewing style. We also conducted 5 focus group discussions with 27 participants, 2 Latinx groups and 3 Black groups (Mage = 61 years). The focus group participants identified as caregivers of cancer survivors.

**Results:**

Thematic analysis resulted in the following themes: culture and dealing with cancer, conflict and control, health care access barriers, and rethinking survivorship in the community. The themes offer insight into sociocultural factors that influence the quality of life of cancer survivors and those in their communities.

**Conclusion:**

To better understand and address issues of cancer health inequity in the country, the findings of this study will offer an opportunity to advance knowledge on sociocultural factors influencing cancer survivorship in Black and Latinx communities.

